# Epidermolysis Bullosa With Pyloric Stenosis: A Novel Lethal Variant

**DOI:** 10.7759/cureus.41167

**Published:** 2023-06-30

**Authors:** Rahaf A Ghazzawi, Alfia Fatma

**Affiliations:** 1 Obstetrics and Gynecology, International Medical Center Hospital, Jeddah, SAU

**Keywords:** itga6, atresia, pyloric, junctional, bullosa, epydemolysis

## Abstract

Epidermolysis bullosa (EB) is a rare and genetically inherited skin fragility disorder causing mucocutaneous blistering, erosion, and ulceration as a result of even minor trauma. Junctional EB (JEB), which is a type of EB, is inherited via an autosomal recessive pattern and characterized by blisters that appear in the lamina lucida of the basement membrane zone, which is the junction between the epidermis and dermis.

The integrin genes (ITGA6, ITGB4) are responsible for the majority of JEB mutations. We present a case of lethal JEB and pyloric atresia with aplasia cutis congenita (ACC), with a homozygous pathogenic variant identified in the ITGA6 gene, c.1688dup. The diagnosis was made by whole exome sequencing (WES) postnatally after consecutive third pregnancy loss in the last trimester in a consanguineous couple.

As these cases have a poor prognosis, genetic counseling, invasive prenatal testing, and preimplantation genetic diagnosis (PGD) have an evolving and indispensable role in the management of future pregnancies.

## Introduction

Epidermolysis bullosa (EB) is a rare, genetically inherited skin fragility disorder, causing mucocutaneous blistering, erosions, and ulceration as a result of even minor trauma [1]. Junctional EB (JEB) which is a type of EB is a skin disorder defined by dermal-epidermal junction fragility [2]. JEB is inherited via autosomal recessive mode. The expression of the α6β4 integrin is altered because of mutations in ITGA6 and ITGB4, which result in a structural defect of the hemidesmosome. These defects of the hemidesmosome result in skin erosions and blistering, as well as urogenital and gastrointestinal tract malformations [3]. In some cases, they can cause a focal or extensive absence of the epidermis, dermis, and, occasionally, subcutaneous tissue, leading to aplasia cutis congenita (ACC) [4]. We present a recurrent case of lethal JEB and pyloric atresia with ACC due to a homozygous pathogenic variant, c.1688dup, identified in the ITGA6 gene.

## Case presentation

A 33-year-old woman, Gravida 3 Para2 +0, live 0, presented to our fetal medicine unit with suspected intrauterine fetal death (IUFD) at 32 weeks. She had no significant medical or surgical history, but the couple had consanguinity.

Her first pregnancy included early-onset intrauterine growth restriction with duodenal atresia, ventricular septal defect, and talipes. The baby was delivered at 34 weeks by cesarean section. At birth, the baby had bullous eruptions and peeling of the skin and died two days after birth. The following year, her second pregnancy ended with IUFD at 32 weeks.

**Figure 1:**
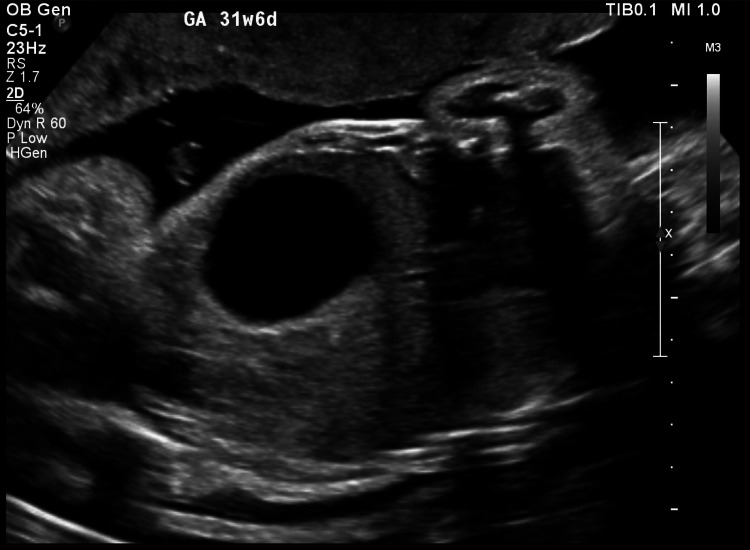
Pyloric atresia

**Figure 2:**
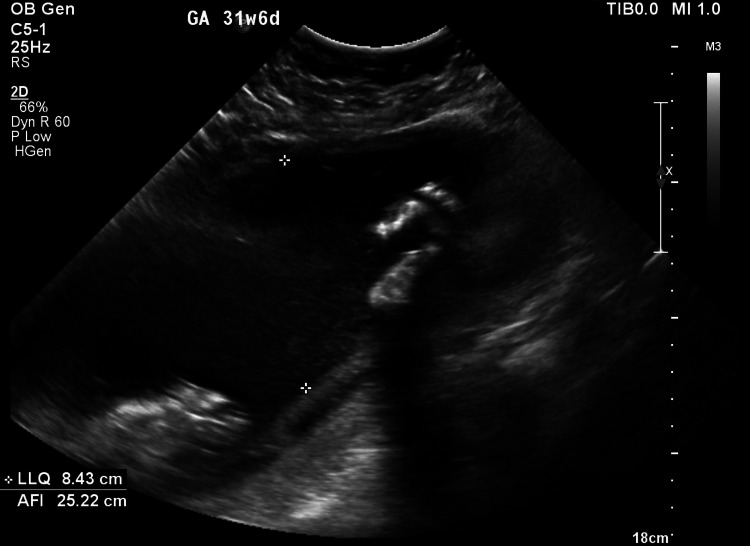
Snowflake sign

In her current pregnancy, she was following up in another hospital and was informed that the morphology scan was normal. Only at 30 weeks was she told that she had polyhydramnios. An ultrasound scan performed in the fetal medicine unit to confirm IUFD showed a severely dilated stomach, indicating pyloric atresia (Figure 1), along with a moderately dilated right renal pelvis (14 mm) and a dilated ureter. The liquor appeared echogenic, suggesting a snowflake sign (Figure 2), with severe polyhydramnios with an amniotic fluid Index of 34 cm.

**Figure 3:**
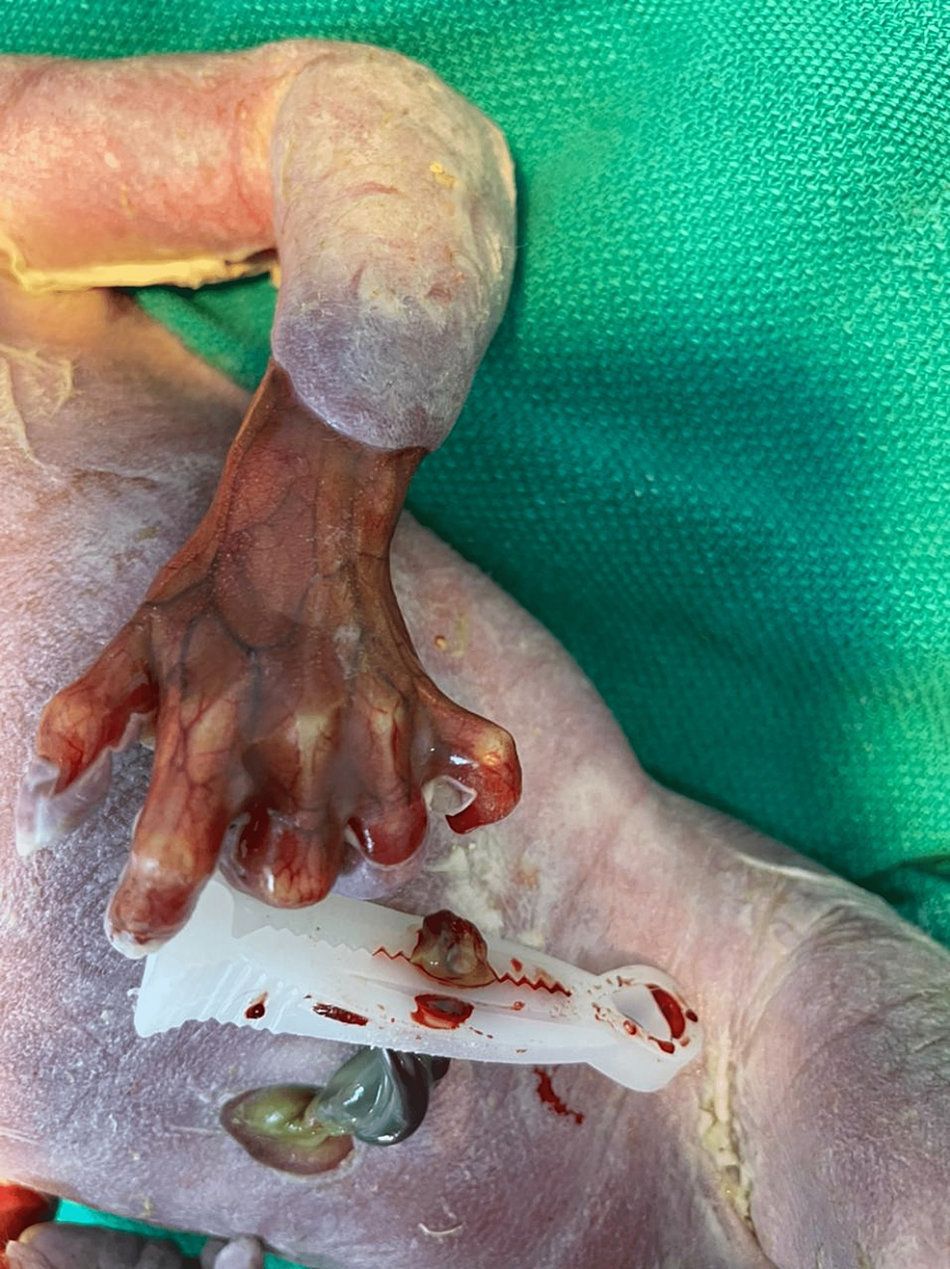
Blistering of the skin

**Figure 4:**
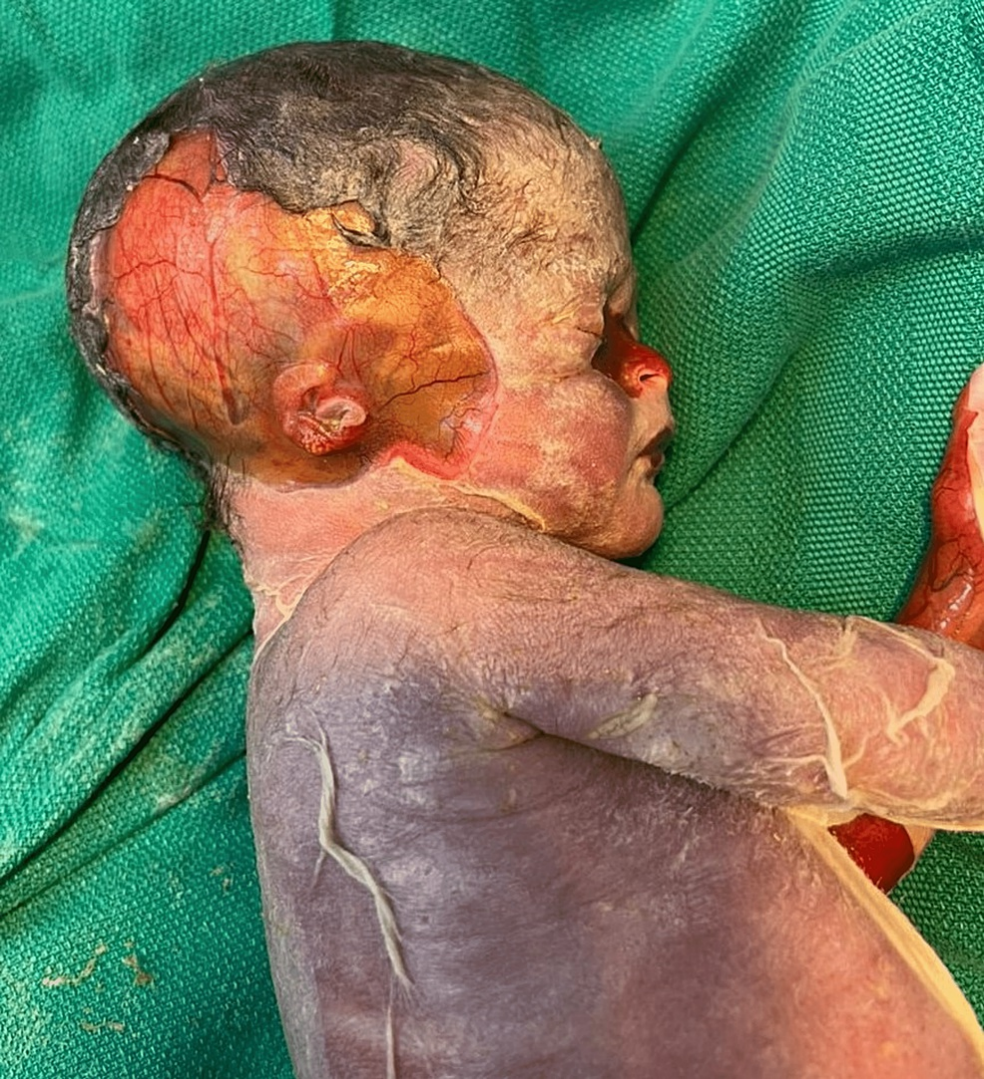
ACC, low-set ears, an absent nasal septal cartilage, and a depressed nasal bridge

The couple received extensive counseling, was informed about the possibility of genetic abnormalities, and was offered postnatal genetic testing. After birth, the fetus showed blistering of the skin (Figure 3), ACC, low-set ears, an absent nasal septal cartilage, and a depressed nasal bridge (Figure 4).

After delivery, a skin biopsy was taken to perform microarrays and whole exome sequencing (WES).

As a result of genetic testing, homozygous likely pathogenic variant c.1688dup, p.(Met563Ilefs*10), was identified in the ITGA6 gene. The result was consistent with a genetic diagnosis of autosomal recessive EB, junctional 6, with pyloric atresia. Postdelivery, the mother informed that her second IUFD also had blistering of the skin, a depressed nasal bridge, and low-set ears. Both parents were tested and found to be carriers of the ITGA6 gene. Following this, the couple received genetic counseling and was offered a preimplantation genetic diagnosis (PGD) for the next pregnancy.

## Discussion

JEB is inherited via an autosomal recessive pattern and characterized by blisters that appear in the lamina lucida of the basement membrane zone, which is the junction between the epidermis and the dermis. The incidence and prevalence of JEB are 2.68 per million live births and 0.49 per million people, respectively [1]. JEB is further classified into three subtypes: Herlitz, nonHerlitz, and JEB with pyloric atresia (JEB-PA)(Carmi syndrome). Swinburne and Kholer first reported an association between pyloric atresia and EB in 1968 [3]. Carmi later described the pathophysiology of JEB-PA; hence, the disease is called Carmi syndrome. Patients with Carmi syndrome exhibit a syndromic conjunction of skin fragility with congenital gastrointestinal atresia, most frequently pyloric, although reports of duodenal atresia also exist [5].

Mutations in ITGA6 and ITGB4 that occur in JEB change the expression of the α6B4 integrin, resulting in a hemidesmosome structural defect. Hemidesmosomes maintain the connection between the dermis and the epidermis by acting as points of attachment for the intermediate filament system. This leads to blisters and erosions on the skin, urogenital disorders, eye disorders, and gastrointestinal tract malformations. Pyloric atresia in EB is also a result of scarring. Dysfunctional or absent hemidesmosomes result in the separation of the epidermis or the intestinal mucosa, with consequent inflammation and fibrosis, leading to obstruction in areas like the pylorus [3,6].

ACC is a rare congenital skin defect characterized by a focal or extensive absence of the epidermis, dermis, and, occasionally, subcutaneous tissue.

The literature shows that JEB-PA-ACC is most often lethal and suggests that ACC is a poor prognostic factor for JEB-PA patients. As a result of skin barrier breakdown, these patients develop septicemia, electrolyte disturbances, a loss of protein, and hypoalbuminemia, making septicemia the most common cause of neonatal death in these cases [3]. Intrauterine mechanical trauma may cause ACC, but the combination of ACC and JEB-PA suggests a common genetic basis for their pathogenesis [1,2]. We believe that if the disease is severe, intrauterine lesions can occur, which may cause ACC. Therefore, JEB-PA with ACC may be at the more severe end of the spectrum.

Prenatal ultrasounds can help screen for JEB-PA, according to a systematic review of 100 articles by Mylonas et al. in 2018 [6]. Prenatal ultrasounds may show signs of urinary, jejunal, or duodenal atresia, gastric dilatation that indicates gastric outlet obstruction, and polyhydramnios. In 1990, Carmi and Meiznerin first identified the "snowflake sign," or the echogenic appearance of the amniotic fluid due to the scaling of fetal skin, in fetuses with EB [5].

Our case had signs suggestive of pyloric stenosis and a snowflake sign on the antenatal ultrasound. As the patient presented late in the pregnancy, after fetal demise, the diagnosis of JEB-PA was only confirmed postnatally by WES of a skin biopsy. The result was consistent with a genetic diagnosis of autosomal recessive EB, junctional 6, with pyloric atresia. A homozygous ITGA6 variant, c.1688dup, was identified.

Mutations in ITGA6 are less frequent than those in ITGB4 [3]. Pathogenic variants in the ITGA6 gene have been associated with autosomal recessive EB, junctional 6, and pyloric atresia (OMIM®: 619817). Skin manifestations include severe blistering, atrophic scarring, and nail dystrophy. A congenital ACC is common, and ear anomalies are also relatively common. Ruzzi et al. in 1997 described the first mutation in the a6 integrin gene in a PA-JEB patient presenting with generalized skin blistering, aplasia cutis, and defective expression of integrin a6b4. The mutation (791delC) was a homozygous deletion of a single base (C) leading to a frameshift and a premature termination codon that results in a complete absence of a6 polypeptide [7].

In this report, the identified ITGA6 variant c.1688dup p.(Met563Ilefs*10) creates a shift in the reading frame starting at codon 563. The new reading frame ends in a stop codon 9 positions downstream. Although the variant of ITGA6, c.1688dup, has been labeled as likely pathogenic by the laboratory due to a lack of other reports in the past, in our case, the three recurrent pregnancy losses most likely stemmed from this variant, as the parents were carriers of this autosomal recessive disorder.

JEB-PA with ACC is known to be an untreatable condition with a poor prognosis. However, identification of the underlying genetic abnormality is critical for providing appropriate genetic counseling, prenatal testing, or PGD. In lethal cases, even if intestinal obstructions are corrected successfully, patients suffer from septicemia, electrolyte imbalance, protein loss, and hypoalbuminemia, which are related to the loss of the skin barrier [3].

Considering the recurrence risk of 25% in any pregnancy with an autosomal recessive condition like JEB, genetic counseling and prenatal testing play a crucial role in the management of affected families. Genetic testing to investigate the specific gene mutation or molecular analysis of the fetal DNA can be performed through chorionic villus sampling or amniocentesis to make a prenatal diagnosis. However, this can have a significant emotional impact on the family if another pregnancy is affected, leading to the termination of the pregnancy [8].

PGD is revolutionary in the management of these cases. It is a highly specialized technique, available in a few centers globally, that involves the genetic screening of cells from oocytes or early embryos before pregnancy. Confirmed, mutation-free oocytes or embryos can be used subsequently for assisted fertilization. In addition to a pregnancy rate of approximately 25%, associated risks of in vitro fertilization, such as ovarian hyperstimulation syndrome and multiple pregnancies, also exist [1]. Hence, early identification of these cases and offering them prenatal diagnosis for confirmation is vital in the management of such rare conditions.

## Conclusions

JEB-PA with ACC is an untreatable condition with a poor prognosis. Considering the ITGA6 mutation is rare and primarily homozygous, with a relatively high incidence of consanguinity, it raises the possibility that the ITGA6 mutation is family specific. Hence, genetic counseling, prenatal diagnosis after spontaneous conception, and PGD play an imperative role in the management of future pregnancies for these couples.
